# Poly[diaqua-μ_4_-biphenyl-4,4′-dicarboxyl­ato-magnesium(II)]

**DOI:** 10.1107/S1600536809002864

**Published:** 2009-01-28

**Authors:** Hsin-Kuan Liu, Xiang-Wen Peng, Chia-Her Lin

**Affiliations:** a Department of Chemistry, Chung-Yuan Christian University, Chung-Li 320, Taiwan

## Abstract

The solvothermal reaction of magnesium nitrate with bi­phenyl-4,4′-dicarboxylic acid in *N*,*N*-dimethyl­formamide and water leads to the formation of crystals of the title complex, [Mg(C_14_H_8_O_4_)(H_2_O)_2_]_*n*_. In the crystal structure, the Mg cations are coordinated by six O atoms from two water mol­ecules and four symmetry-related biphenyl-4,4′-dicarboxyl­ate anions within slightly distorted octa­hedra. The Mg cations are located on a center of inversion, the biphenyl-4,4′-dicarboxyl­ate anions around a twofold rotation axis and the water mol­ecule in a general position. The Mg cations are linked by the anions into a three-dimensional framework.

## Related literature

For related structures, see: Kitagawa *et al.* (2004 ▶).
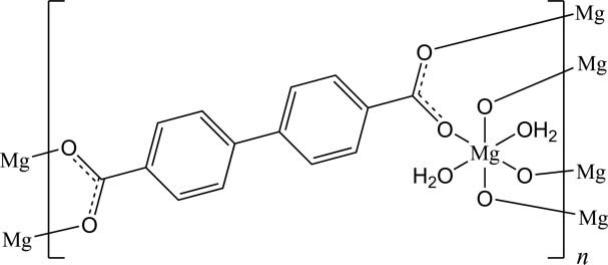


## Experimental

### 

#### Crystal data

[Mg(C_14_H_8_O_4_)(H_2_O)_2_]
*M* *_r_* = 150.27
Orthorhombic, 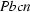

*a* = 6.5913 (10) Å
*b* = 7.2900 (9) Å
*c* = 26.759 (4) Å
*V* = 1285.8 (3) Å^3^
*Z* = 8
Mo *K*α radiation
μ = 0.16 mm^−1^
*T* = 295 (2) K
0.15 × 0.10 × 0.05 mm

#### Data collection

Bruker APEXII CCD diffractometer
Absorption correction: multi-scan (*SADABS*; Bruker, 2007 ▶) *T*_min_ = 0.976, *T*_max_ = 0.995
5950 measured reflections
1589 independent reflections
1048 reflections with *I* > 2σ(*I*)
*R*_int_ = 0.048

#### Refinement

*R*[*F* ^2^ > 2σ(*F* ^2^)] = 0.041
*wR*(*F* ^2^) = 0.106
*S* = 1.02
1589 reflections
97 parameters
H-atom parameters constrained
Δρ_max_ = 0.30 e Å^−3^
Δρ_min_ = −0.30 e Å^−3^

### 

Data collection: *APEX2* (Bruker, 2007 ▶); cell refinement: *SAINT* (Bruker, 2007 ▶); data reduction: *SAINT*; program(s) used to solve structure: *SHELXS97* (Sheldrick, 2008 ▶); program(s) used to refine structure: *SHELXL97* (Sheldrick, 2008 ▶); molecular graphics: *SHELXTL* (Sheldrick, 2008 ▶); software used to prepare material for publication: *SHELXTL*.

## Supplementary Material

Crystal structure: contains datablocks I, global. DOI: 10.1107/S1600536809002864/nc2132sup1.cif

Structure factors: contains datablocks I. DOI: 10.1107/S1600536809002864/nc2132Isup2.hkl

Additional supplementary materials:  crystallographic information; 3D view; checkCIF report

## Figures and Tables

**Fig. 1.**
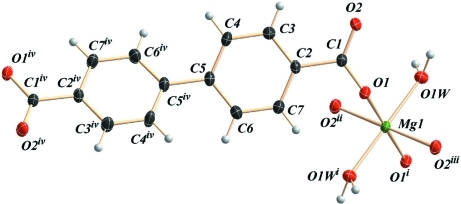
Crystal structure of the title compound with labeling and displacement ellipsoids drawn at the 50% probability level. [symmetry codes: (i) -x + 2,-y + 1,-z; (ii) -x + 3/2,y - 1/2,z; (iii) x + 1/2,-y + 3/2,-z; (iv) -x + 2,y,-z + 1/2.].

**Fig. 2.**
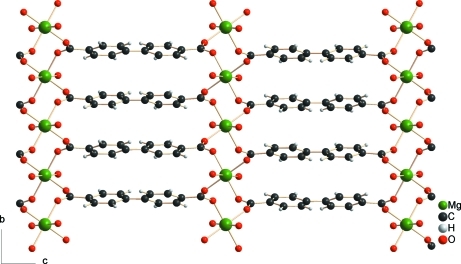
Crystal structure of the title compound with view along the a axis.

## supplementary crystallographic information

### Comment

The synthesis of coordination polymers, or so-called metal-organic frameworks (MOF), has been a subject of intense research owing to their interesting structural chemistry and potential applications in gas storage, separation, catalysis, magnetism, luminescence. A large number of these compounds have been synthesized by solvothermal reactions with organic carboxyl acids (Kitagawa *et al.*, 2004). Here we report on the new metal organic framework bis(aqua)-biphenyl-4,4'-dicarboxylate magnesium (II). In the crystal structure the Mg cations are sorrounded by two O atoms from two symmetry related water molecules and four O atoms of four symmetry related anions (Fig. 1). The coordination polyhedron around the Mg cations can be described as a slightly distorted octahedron. The Mg cations are linked *via* the anions into a three-dimensional network (Fig. 2).

### Experimental

The reaction was carried out under solvothermal conditions in a teflon-lined autoclav with an inner volume of 23 ml. A single-phase product consisting of transparent colorless crystals was obtained by heating a mixture of Mg(NO_3_)_2_˙6H_2_O, (0.1281 g, 0.5 mmol), biphenyl-4,4'-dicarboxylic acid (C_14_H_10_O_4_, 0.0290 g, 0.125 mmol), *N*,*N*-dimethylformamide (10.0 ml), and H_2_O (2.0 ml)at 423 K for 2 d followed by slow cooling at 6 K/h to room temperature.

### Refinement

The C—H H atoms were positioned with idealized geometry and were refined isotropic using a riding model with C—H = 0.93 Å and *U*_iso_(H) = 1.2*U*_eq_(C). The H atoms at the water molecule were found in difference map and were refined with varying coordinates isotropic.

### Crystal data

**Table:**

[Mg(C_14_H_8_O_4_)(H_2_O)_2_]	*D*_x_ = 1.553 Mg m^−^^3^
*M**_r_* = 150.27	Mo *K*α radiation, λ = 0.71073 Å
Orthorhombic, *P**b**c**n*	Cell parameters from 1007 reflections
*a* = 6.5913 (10) Å	θ = 3.1–25.2°
*b* = 7.2900 (9) Å	µ = 0.16 mm^−^^1^
*c* = 26.759 (4) Å	*T* = 295 K
*V* = 1285.8 (3) Å^3^	Lamellar, colorless
*Z* = 8	0.15 × 0.10 × 0.05 mm
*F*(000) = 624	

### Data collection

**Table:**

Bruker APEXII CCD diffractometer	1589 independent reflections
Radiation source: fine-focus sealed tube	1048 reflections with *I* > 2σ(*I*)
graphite	*R*_int_ = 0.048
φ and ω scans	θ_max_ = 28.4°, θ_min_ = 1.5°
Absorption correction: multi-scan (*SADABS*; Bruker, 2007)	*h* = −7→8
*T*_min_ = 0.976, *T*_max_ = 0.995	*k* = −9→9
5950 measured reflections	*l* = −22→35

### Refinement

**Table:**

Refinement on *F*^2^	Primary atom site location: structure-invariant direct methods
Least-squares matrix: full	Secondary atom site location: difference Fourier map
*R*[*F*^2^ > 2σ(*F*^2^)] = 0.041	Hydrogen site location: inferred from neighbouring sites
*wR*(*F*^2^) = 0.106	H-atom parameters constrained
*S* = 1.01	*w* = 1/[σ^2^(*F*_o_^2^) + (0.0448*P*)^2^ + 0.3401*P*] where *P* = (*F*_o_^2^ + 2*F*_c_^2^)/3
1589 reflections	(Δ/σ)_max_ < 0.001
97 parameters	Δρ_max_ = 0.30 e Å^−^^3^
0 restraints	Δρ_min_ = −0.30 e Å^−^^3^

### Special details

**Table:**

Geometry. All e.s.d.'s (except the e.s.d. in the dihedral angle between two l.s. planes) are estimated using the full covariance matrix. The cell e.s.d.'s are taken into account individually in the estimation of e.s.d.'s in distances, angles and torsion angles; correlations between e.s.d.'s in cell parameters are only used when they are defined by crystal symmetry. An approximate (isotropic) treatment of cell e.s.d.'s is used for estimating e.s.d.'s involving l.s. planes.
Refinement. Refinement of *F*^2^ against ALL reflections. The weighted *R*-factor *wR* and goodness of fit *S* are based on *F*^2^, conventional *R*-factors *R* are based on *F*, with *F* set to zero for negative *F*^2^. The threshold expression of *F*^2^ > σ(*F*^2^) is used only for calculating *R*-factors(gt) *etc*. and is not relevant to the choice of reflections for refinement. *R*-factors based on *F*^2^ are statistically about twice as large as those based on *F*, and *R*- factors based on ALL data will be even larger.

### Fractional atomic coordinates and isotropic or equivalent isotropic displacement parameters (Å^2^)

**Table:**

	*x*	*y*	*z*	*U*_iso_*/*U*_eq_	
Mg1	1.0000	0.5000	0.0000	0.0159 (2)	
C1	0.7888 (3)	0.7850 (2)	0.06884 (7)	0.0162 (4)	
C2	0.8503 (3)	0.7715 (3)	0.12262 (7)	0.0184 (4)	
C3	0.7124 (3)	0.8132 (3)	0.16000 (7)	0.0245 (5)	
H3	0.5796	0.8440	0.1517	0.029*	
C4	0.7715 (4)	0.8092 (3)	0.20963 (7)	0.0259 (5)	
H4	0.6775	0.8380	0.2343	0.031*	
C5	0.9691 (3)	0.7629 (3)	0.22334 (7)	0.0211 (5)	
C6	1.1051 (3)	0.7183 (3)	0.18550 (7)	0.0261 (5)	
H6	1.2372	0.6854	0.1938	0.031*	
C7	1.0476 (3)	0.7220 (3)	0.13581 (8)	0.0242 (5)	
H7	1.1409	0.6915	0.1111	0.029*	
O1	0.9201 (2)	0.74253 (17)	0.03552 (5)	0.0194 (3)	
O1W	0.7228 (2)	0.48848 (18)	−0.03619 (5)	0.0221 (3)	
H1WA	0.6306	0.5799	−0.0412	0.080*	
H1WB	0.6335	0.4082	−0.0293	0.080*	
O2	0.6164 (2)	0.8454 (2)	0.05857 (5)	0.0233 (4)	

### Atomic displacement parameters (Å^2^)

**Table:**

	*U*^11^	*U*^22^	*U*^33^	*U*^12^	*U*^13^	*U*^23^
Mg1	0.0166 (5)	0.0205 (4)	0.0107 (4)	0.0010 (4)	0.0010 (4)	−0.0006 (4)
C1	0.0195 (11)	0.0161 (9)	0.0129 (9)	0.0000 (8)	0.0000 (8)	0.0002 (7)
C2	0.0214 (12)	0.0218 (10)	0.0121 (9)	0.0017 (8)	−0.0020 (8)	0.0004 (8)
C3	0.0214 (12)	0.0352 (12)	0.0171 (10)	0.0075 (10)	−0.0001 (9)	0.0004 (9)
C4	0.0271 (13)	0.0388 (11)	0.0116 (9)	0.0057 (10)	0.0022 (9)	−0.0003 (9)
C5	0.0240 (12)	0.0272 (10)	0.0120 (10)	0.0006 (9)	−0.0017 (9)	−0.0002 (8)
C6	0.0202 (12)	0.0416 (12)	0.0166 (10)	0.0029 (10)	−0.0037 (9)	0.0012 (9)
C7	0.0216 (12)	0.0369 (12)	0.0141 (10)	0.0042 (10)	0.0010 (8)	−0.0005 (9)
O1	0.0221 (8)	0.0232 (7)	0.0129 (7)	0.0025 (6)	0.0012 (6)	−0.0015 (6)
O1W	0.0165 (8)	0.0279 (7)	0.0221 (7)	0.0001 (6)	−0.0011 (6)	0.0012 (6)
O2	0.0200 (8)	0.0346 (8)	0.0152 (7)	0.0065 (7)	−0.0015 (7)	0.0041 (6)

### Geometric parameters (Å, °)

**Table:**

Mg1—O1W^i^	2.0696 (14)	C3—H3	0.9300
Mg1—O1W	2.0696 (14)	C4—C5	1.395 (3)
Mg1—O1	2.0753 (12)	C4—H4	0.9300
Mg1—O1^i^	2.0753 (12)	C5—C6	1.391 (3)
Mg1—O2^ii^	2.0774 (13)	C5—C5^iv^	1.484 (4)
Mg1—O2^iii^	2.0774 (13)	C6—C7	1.383 (3)
C1—O2	1.249 (2)	C6—H6	0.9300
C1—O1	1.281 (2)	C7—H7	0.9300
C1—C2	1.498 (3)	O1W—H1WA	0.9119
C2—C3	1.385 (3)	O1W—H1WB	0.8502
C2—C7	1.395 (3)	O2—Mg1^v^	2.0774 (13)
C3—C4	1.384 (3)		
			
O1W^i^—Mg1—O1W	180.00 (10)	C4—C3—C2	120.2 (2)
O1W^i^—Mg1—O1	88.58 (5)	C4—C3—H3	119.9
O1W—Mg1—O1	91.42 (5)	C2—C3—H3	119.9
O1W^i^—Mg1—O1^i^	91.42 (5)	C3—C4—C5	121.3 (2)
O1W—Mg1—O1^i^	88.58 (5)	C3—C4—H4	119.3
O1—Mg1—O1^i^	180.00 (6)	C5—C4—H4	119.3
O1W^i^—Mg1—O2^ii^	89.72 (5)	C6—C5—C4	117.84 (18)
O1W—Mg1—O2^ii^	90.28 (5)	C6—C5—C5^iv^	121.5 (2)
O1—Mg1—O2^ii^	91.31 (5)	C4—C5—C5^iv^	120.6 (2)
O1^i^—Mg1—O2^ii^	88.69 (5)	C7—C6—C5	121.3 (2)
O1W^i^—Mg1—O2^iii^	90.28 (5)	C7—C6—H6	119.4
O1W—Mg1—O2^iii^	89.72 (5)	C5—C6—H6	119.4
O1—Mg1—O2^iii^	88.69 (5)	C6—C7—C2	120.2 (2)
O1^i^—Mg1—O2^iii^	91.31 (5)	C6—C7—H7	119.9
O2^ii^—Mg1—O2^iii^	180.00 (8)	C2—C7—H7	119.9
O2—C1—O1	123.14 (17)	C1—O1—Mg1	134.15 (12)
O2—C1—C2	118.73 (17)	Mg1—O1W—H1WA	128.9
O1—C1—C2	118.03 (17)	Mg1—O1W—H1WB	122.5
C3—C2—C7	119.06 (18)	H1WA—O1W—H1WB	94.2
C3—C2—C1	120.10 (18)	C1—O2—Mg1^v^	133.89 (13)
C7—C2—C1	120.82 (18)		

Symmetry codes: (i) −*x*+2, −*y*+1, −*z*; (ii) −*x*+3/2, *y*−1/2, *z*; (iii) *x*+1/2, −*y*+3/2, −*z*; (iv) −*x*+2, *y*, −*z*+1/2; (v) *x*−1/2, −*y*+3/2, −*z*.
